# The Role of Host Genetics in Susceptibility to Influenza: A Systematic Review

**DOI:** 10.1371/journal.pone.0033180

**Published:** 2012-03-15

**Authors:** Peter Horby, Nhu Y. Nguyen, Sarah J. Dunstan, J. Kenneth Baillie

**Affiliations:** 1 Oxford University Clinical Research Unit–Wellcome Trust Major Overseas Programme, Hanoi, Vietnam; 2 The Roslin Institute, University of Edinburgh, Edinburgh, United Kingdom; McMaster University, Canada

## Abstract

**Background:**

The World Health Organization has identified studies of the role of host genetics on susceptibility to severe influenza as a priority. A systematic review was conducted to summarize the current state of evidence on the role of host genetics in susceptibility to influenza (PROSPERO registration number: CRD42011001380).

**Methods and Findings:**

PubMed, Web of Science, the Cochrane Library, and OpenSIGLE were searched using a pre-defined strategy for all entries up to the date of the search. Two reviewers independently screened the title and abstract of 1,371 unique articles, and 72 full text publications were selected for inclusion. Mouse models clearly demonstrate that host genetics plays a critical role in susceptibility to a range of human and avian influenza viruses. The *Mx* genes encoding interferon inducible proteins are the best studied but their relevance to susceptibility in humans is unknown. Although the *MxA* gene should be considered a candidate gene for further study in humans, over 100 other candidate genes have been proposed. There are however no data associating any of these candidate genes to susceptibility in humans, with the only published study in humans being under-powered. One genealogy study presents moderate evidence of a heritable component to the risk of influenza-associated death, and while the marked familial aggregation of H5N1 cases is suggestive of host genetic factors, this remains unproven.

**Conclusion:**

The fundamental question “Is susceptibility to severe influenza in humans heritable?” remains unanswered. Not because of a lack of genotyping or analytic tools, nor because of insufficient severe influenza cases, but because of the absence of a coordinated effort to define and assemble cohorts of cases. The recent pandemic and the ongoing epizootic of H5N1 both represent rapidly closing windows of opportunity to increase understanding of the pathogenesis of severe influenza through multi-national host genetic studies.

## Introduction

The on-going family clustering of highly pathogenic avian influenza A/H5N1 cases, as demonstrated by the deaths in 2011 of a mother and son in Cambodia, and of two siblings and their mother in Indonesia, has led to much speculation that host genetics play a critical role in susceptibility to H5N1 influenza [1]-[5]. Although H5N1 is an unusually virulent influenza virus, patterns of disease in other influenza epidemics also suggest a possible role for host genetics in susceptibility to severe influenza: around one-quarter to one-half of patients with severe pandemic influenza A/H1N1/09 were previously healthy, with no co-existing medical condition or other predisposing factors [6]. Whilst the viral genetic determinants of influenza severity have been intensively studied, host determinants are much less well studied.

A better understanding of the biological predispositions and pathways leading to severe influenza may lead to improved therapeutic options, and in 2009 the World Health Organization identified studies of the role of host genetic factors on susceptibility to severe influenza as a priority [7], [8]. This systematic review was conducted with the objective of summarizing the current state of evidence that host genetic factors play a role in human susceptibility to influenza virus infection or disease.

## Methods

The systematic review was conducted and reported in accordance with the PRISMA guidelines and the protocol was registered on the international prospective register of systematic reviews (PROSPERO registration number: CRD42011001380. Available at: http://www.crd.york.ac.uk/prospero/). Briefly, we conducted a systematic review to summarize relevant published and unpublished evidence of host genetic factors influencing the risk of influenza infection or disease (illness following infection). This comprised a search of PubMed, Web of Science, the Cochrane Library, and OpenSIGLE (grey literature bibliographic database) using a pre-defined search strategy. The full systematic review protocol, including the search strategy, is shown in the Supporting Information file S1. Two reviewers independently screened all the titles and abstracts to identify publications that may be relevant. A third reviewer assessed the two independent lists of selected and rejected sources and made the final selection where there were discrepancies. The full text of all the sources in the final list was obtained and reviewed independently by two reviewers to decide if they met the inclusion/exclusion criteria. The reference list of all selected sources was reviewed to identify relevant articles that may have been missed by the search strategy. Individual researchers were contacted directly to obtain additional information where the source material could not be obtained or to enquire about on-going or unpublished research.

Certain categories of research were excluded from this review. A very large number of genes are up or down regulated during influenza infection and disease, and it was deemed outside the scope of this piece of work to review the extensive literature on the biological responses to natural or experimental influenza infection. These studies have recently been reviewed elsewhere [9]. Therefore we excluded studies of the molecular biology and pathogenesis of influenza except where the study directly compared the response to infection in genetically distinct animal strains with the objective of identifying host genetic determinants of response. We also excluded studies that solely examined the affects of gene knockouts, since a knockout mouse phenotype, although very useful for understanding pathogenesis, does not provide information on heritability of susceptibility under normal conditions, representing null alleles which rarely occur as such in the human population.

## Results

**Figure 1:**
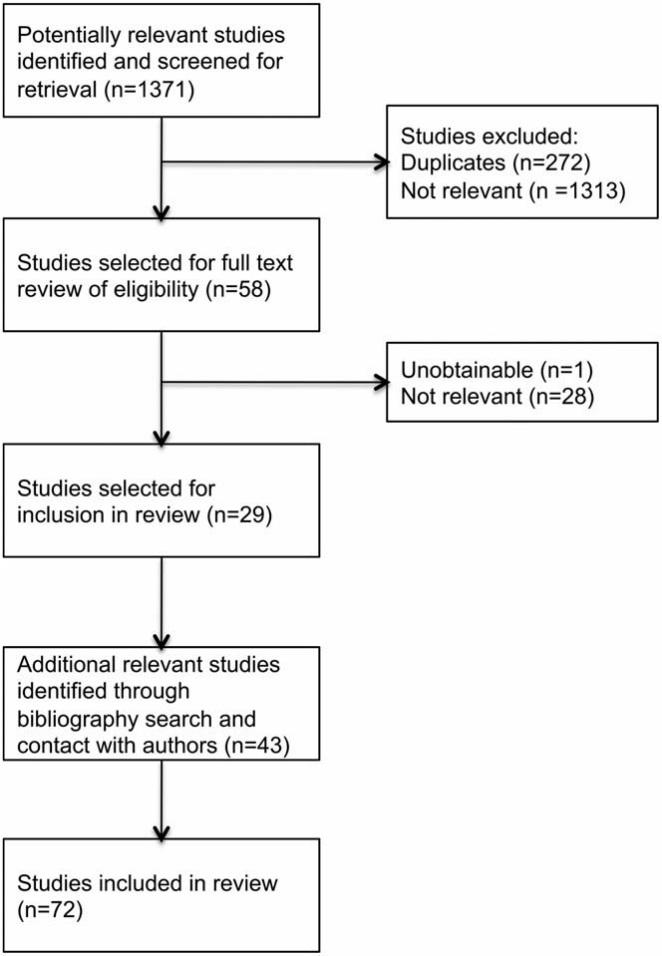
Identification and screening of articles for inclusion in systematic review.

The search strategy was run on 26^th^ June 2011 and identified 1371 unique articles published in English for which the title and abstract was reviewed. 58 met the criteria for full text review, of which 29 were considered relevant to the study and could be obtained (Figure 1). A further 43 relevant articles were identified through a review of the bibliographies of the 29 selected papers and through contact with lead authors. A total of 72 articles were therefore included in the review. The identified published evidence fell into the following categories: studies in animals of host genetics; studies or reports of familial aggregation or heritability; studies in humans of blood group; studies in humans of HLA type; and studies in humans of host genetics. Key studies of heritability or genetic susceptibility in mice are shown in Table 1, whilst key studies of familial aggregation, heritability, or genetic susceptibility in humans are summarised in Table 2.

**Table 1:** Key studies of heritability or genetic susceptibility in mice.

Author (Year)	Study/Investigation	Main Findings
Mx		
Lindenmann J (1962) [10]	Experimental inoculation of A2G mice with H1N1/NWS/1933 virus	A2G mice exhibit considerable resistance to intracerebral and intranasal to H1N1/NWS/1933 inoculation.
Staeheli P (1988) [18]	Molecular analysis of *Mx1* alleles using restriction fragment length polymorphism (RFLP) and southern blot analysis of classical inbred mouse strains.	The establishment of *Mx1* ^+^ and *Mx1* ^-^ mouse lines was due to a single nonsense mutation in the *Mx* gene, which was represented in present-day mice by the prototype strains A2G and CBA/J.
Horisberger MA (1995) [94]	Review article of *Mx* genes and influenza	
Salomon R (2007) [23]	Comparison of the effect in mice with and without a functional *Mx1* gene of inoculation with H5N1 A/Vietnam/ 1203/04 and reassortants with the non-lethal virus A/chicken/ Vietnam/C58/04.	Compared to *Mx1* ^-/-^ mice, *Mx1* ^+/+^ mice were protected from A/Vietnam/1203/04, showing lower viral tires, less pathology, and no deaths.
Tumpey TM (2007) [24]	Comparison of the effect in mice with and without a functional *Mx1* gene of inoculation with H1N1/1918 and H5N1 A/Vietnam/1203/04.	Compared to *Mx1* ^-/-^ mice, *Mx1* ^+/+^ mice were protected from 1918 H1N1 and A/Vietnam/1203/04, showing lower viral tires, less weight loss, and fewer deaths.
Grimm D (2007) [25]	Characterization of influenza A H1N1 (PR8) that is unusually virulent in *Mx1* ^+/+^ mice.	Virulence of PR8 is due to high replication ability, not inherent resistance to Mx1.
Dittmann J (2008) [26]	In-vitro study of the inhibitory effect of mouse Mx1 protein and human MxA protein on different influenza strains in cell culture or minireplicon assay.	Influenza A viruses varied in their sensitivity to Mx proteins, with avian virus showing greater sensitivity than human viruses.
Haller O (2009) [22]	Review article of *Mx* genes and influenza	
Zimmermann P (2011) [28]	Study of the inhibitory effect of mouse Mx1 protein and human MxA protein on H1N1/09 (A/Hamburg/4/09) and highly pathogenic avian H5N1 (A/Thailand/1(KAN-1)/04)	H5N1 (A/Thailand/1(KAN-1)/04) was more sensitive to Mx proteins than H1N1/09 (A/Hamburg/4/09). This sensitivity was associated with the NP gene.
**Other susceptibility loci**		
Toth LA (1999) [34]	Study of strain associated variation in slow-wave-sleep patterns in response to influenza H3N2 (HK-X31) infection. Sleep measurement of 13 recombinant inbred strains, which were from a cross between C57BL/6ByJ and BALB/cByJ mice. Quantitative Trait Loci (QTL) linked to phenotype were identified using a genome wide linkage scan against 223 loci.	A 10- to 12-cM interval on chromosome 6 between *D6Mit74* and *D6Mit188* contains a quantitative trait loci (QTL) affecting the SWS response to influenza infection during the light phase.
Ding M (2008) [35]	Complementary DNA microarray analysis of lung and basal forebrain of influenza H3N2 (HK-X31) infected and uninfected BALB/cByJ and C57BL/6J mice	In lung, 361 different genes changed expression after influenza infection of BALB/cByJ mice as compared with 16 in C57BL/6J mice. Of 75 genes related to the immune response, 3 showed increased expression in the lungs of infected C57BL/6J mice, compared with 70 in infected BALB/cByJ mice.
Trammell RA (2008) [89]	Review article of human and animal data on host genetic susceptibility to influenza.	
Srivastava B (2009) [31]	Comparison of response to H1N1 (PR8) infection in seven inbred laboratory mouse strains. Additional comparison of response to H7N7 (SC35M) infection in one of the susceptible strains (DBA/2J) and one of the more resistant strains (C57BL/6J).	Different strains exhibited large differences in their response to PR8 infection. DBA/2J mice were highly susceptible to both H1N1 (PR8) and H7N7 (SC35M) infection compared to C57BL/6J mice. DBA/2J mice showed higher viral loads, higher cytokine and chemokine expression, and greater lung pathology compared to C57BL/6J mice.
Boon AC (2009) [32]	Comparison of response of susceptible (DBA/2J) and resistant (C57BL/6J) mice, and 66 recombinant inbred mouse strains to H5N1 (HK213) infection using genome-wide linkage analysis and RNA expression analysis. HK213 was selected for its reduced lethality in C57BL/6J mice while retaining lethality in DBA/2J mice.	Following HK213 infection susceptible strains showed greater viral loads and pro-inflammatory cytokines than resistant strains. Gene mapping revealed five Quantitative Trait Loci located on Chromosomes 2, 7, 11, 15, and 17 associated with resistance to HK213 virus. 121 unique candidate genes were identified whose genetic polymorphisms or different expression levels may have affect H5N1 pathogenesis.
Alberts R (2010) [36]	Comparison of response to H1N1 (PR8) infection in susceptible (DBA/2J) versus resistant (C57BL/6J) mouse strains, analyzed by microarray gene expression analysis.	DBA/2J mice had a stronger chemokine/cytokine response. Innate immune response genes were up regulated in both strains but to a greater extent in the susceptible strain, and overall a large number of genes were up or down regulated only in the susceptible strain.
Boon AC (2011) [39]	Comparison of viral loads and host responses in 21 inbred mouse strains infected with H5N1 (HK213). RNA expression and chemokine/cytokine analysis was undertaken in three susceptible strains (DBA/2S, 129/SvImS, and A/JS) and three resistant strains (SMR, C57BL/6R, and BALB/cR).	Susceptible strains exhibited higher viral loads and concentrations of proinflammatory mediators and expression of proinflammatory genes compared to resistant strains. Relationship between viral load and cytokine concentrations was the same in resistant and susceptible strains.
Trammell RA (2011) [33]	Evaluation of survival, viral load, and cytokine/chemokine responses in lung of four inbred mouse strains (BALB/cByJ, C57BL/6J, A/J, and DBA/2J) and QTL mapping 21 recombinant inbred strains following exposure to H3N2 (HK-X31).	DBA/2J mice demonstrated greater susceptibility to severe disease. There were variable response patterns of mouse strains after in vivo and in vitro exposure to HK-X31. No significant QTL were detected.
Blazejewska P (2011) [27]	Comparison between DBA/2J and C57BL/6J mice of infection with three mouse-adapted variants of the H1N1 PR8 strain: PR8M, PR8F and hvPR8.	The PR8F and the hvPR8 variants were lethal for both DBA/2J and C57BL/6J mouse strains; however, the PR8M variant is only lethal for DBA/2J mice. Infection of C57BL/6J mice with a re-assorted PR8 virus demonstrated that the HA gene is the primary determinant of virulence of the PR8F variant.

**Table 2:** Key studies of familial aggregation, heritability, or genetic susceptibility in humans.

Author (Year)	Study/Investigation	Main Findings
Albright FS (2008) [41]	Study of 4855 deaths from influenza between 1904 and 2004 in a Utah genealogical database.	Evidence of heritability included: risk of influenza death greater in relatives of people who died of influenza than in relatives of the spouse of the person dying of influenza. Deaths in related people frequently did not occur close in time. Greater 'relatedness' amongst influenza deaths compared to age, gender and location matched controls.
Gottfredsson M (2008) [42]	Study of 455 deaths from 1918 influenza over a six-week period in Iceland.	Familial aggregation of deaths was observed but there was no detectable heritable component as the difference in the risk of death between relatives of people who died of influenza and relatives of their spouse was not statistically significant.
Mubareka S (2008) [93]	Commentary on the two genealogy studies	Heritability is unproven but the high risk in spouses identified in both studies indicates that people who share households with severe influenza cases are themselves at increased risk of severe influenza.
Pitzer VE (2007) [53]	Analysis of family clustering of H5N1 cases	A high proportion of household clusters would be expected to be limited to ‘blood relatives’ by chance alone.
Horby P (2010) [3]	Review of epidemiology of H5N1 cases	Epidemiological patterns that suggest host genetic susceptibility include familial aggregation of cases, related cases occurring separated by time and place, and low apparent risk in people who are highly exposed.
Olsen S (2005) [52]	Summary of family clustering of H5N1 cases	15 H5N1 clusters occurring between December 2003 and July 2005 were summarised.
WHO (2011) [5]	Summary of H5N1 clusters reported to WHO, January 2003-March 2009	Amongst a total of 480 Human H5N1 cases reported to WHO there were 54 clusters involving 138 cases (29% of cases). The remaining 342 cases were sporadic. In 50 clusters everyone was a blood relative. In the 4 remaining clusters, 2 clusters that included >3 people, 9/11 people were blood relatives; and in 2 clusters, each contained 2 unrelated people.
Zhang L (2009) [9]	Review of candidate genes for influenza disease and immunity.	Proposed a list of around 100 candidate genes based on published literature of their potential role in the pathogenesis of influenza.
Zuniga J (2011) [88]	Case-control genetic association study. 91 cases of A/H1N1/2009 associated pneumonia and 98 exposed but asymptomatic household contacts. Genotyped using a cardiovascular disease chip with around 50,000 SNPs.	Four SNPs were associated with severe pneumonia with a p<0.0001 after adjustment for gender and comorbidities (obesity, hypertension, and diabetes).

### Animal studies

It has long been known that susceptibility to influenza varies between inbred mouse strains because most laboratory strains carry a mutation in the *Mx1* gene, which is a strong resistance locus for mouse-adapted influenza strains [10]. But more recently, it has been shown that genetic background also plays an important role for resistance or susceptibility, independent of the *Mx1* allele.

#### Myxovirus resistance gene

The resistance of certain inbred mouse strains to influenza A infection was first reported in 1962 [10] and was subsequently localised to the *Mx1* gene on chromosome 16 [11]. The *Mx1* and *Mx2* genes encode interferon inducible proteins, and Mx1 is able to inhibit influenza virus replication [12]-[21]. The role of Mx proteins in protection against influenza has recently been reviewed [22]. Susceptible mice have either deletions or a nonsense point mutation in the *Mx1* gene that results in non-functional Mx1 protein [18]. Mice expressing *Mx1* are also better protected from the high mortality caused by the lethal H5N1 (A/Vietnam/1203/04) and H1N1/1918 viruses and from the lung pathology mediated by these viruses [23], [24]. Influenza viruses differ in their susceptibility to the action of Mx, with adaptive mutations permitting evasion of the Mx response or rapid viral replication outpacing the Mx response [22], [24]-[28]. The H1N1/1918 and H5N1 (A/Vietnam/1203/04) viruses both demonstrate high replication efficiency and are highly pathogenic, and although both are sensitive to the antiviral activity of Mx, H5N1 (A/Vietnam/1203/04) is more sensitive than H1N1/1918 [23], [24], [26]. Influenza virus strains of avian origin appear to have greater sensitivity to Mx than human influenza strains, indicating that adaptive mutations to escape Mx control may be required for successful cross-species transmission [26], [28].

*Mx* gene homologues are found in many species and the homologue in humans is the MxA protein encoded by the *MxA* gene on chromosome 21 [29], [30]. In humans MxA demonstrates antiviral activity [13], [16], [17], [19]-[21] and whilst polymorphisms of the human *MxA* gene exist, their relevance to influenza susceptibility has not been examined.

#### Other susceptibility loci

Although *Mx* genes are the best studied, there are many other candidates genes for influenza susceptibility. Several groups have directly studied the influence of genetic background on the susceptibility of different mouse strains to influenza. All groups confirm that host genetic background plays a critical role in susceptibility to influenza and that highly susceptible mouse strains develop high viral loads, an elevated inflammatory response, and severe lung pathology following infection with a range of influenza viruses [27], [31]-[33]. These studies were performed on inbred mouse strains that carried an *Mx1* mutant allele.

Toth *et al* have examined the genetic basis of differences between mouse strains (BALB/cByJ and C57BL/6J) in sleep patterns during influenza H3N2 A/Hong Kong/X31/68 (HK-X31) infection, identifying a quantitative trait loci (QTL) on chromosome 6 associated with influenza-induced slow-wave sleep patterns [34]. The group also showed large differences between mouse strains (BALB/cByJ and C57BL/6J) in the expression of genes in the lung following influenza HK-X31 infection [35]. In 2011 the same group showed significant strain differences in disease severity (as measured by survival and body temperature), viral titres and cytokine and chemokine concentrations in the lungs of four inbred strains of mice (BALB/cByJ, C57BL/6J, A/J, and DBA/2J) but did not demonstrate any statistically significant genetic loci associated with influenza HK-X31 severity using a QTL approach, although suggestive statistical associations were reported for regions on three chromosomes (G-CSF chromosomes 5; CXCL10 chromosome 9, and IL-6 and CXCL1 on chromosome 18) [33].

Srivastava *et al* examined the susceptibility of seven inbred strains to influenza H1N1 A/Puerto Rico/8/34 (PR8) and identified one resistant (C57BL/6J) and one highly susceptible strain (DBA/2J) [31]. The response of these two strains to H7N7 A/Seal/Massachussetts/1/80 (SC35M) was also examined and DBA/2J mice were highly susceptible to SC35M virus infection compared to C57BL/6J mice. A cross between these two strains showed the resistant phenotype, although with a slightly higher weight loss than the parental resistant strain, suggesting that susceptibility in mice may be a polygenic trait. Further studies by this group examined differential gene expression following PR8 infection of susceptible versus resistant mouse strains [36]. Innate immune response genes were up regulated in both strains but to a greater extent in the susceptible strain, and overall a large number of genes were up or down regulated only in the susceptible strain (75, 538, and 993 on days 1, 2, and 3 after infection respectively). Blazejewska *et al* then looked at the effect of three mouse adapted H1N1 PR8 viruses (“low pathogenic” PR8M and PR8F, and “highly virulent” hvPR8) in two mouse strains that had previously been shown to be resistant (C57BL/6) and susceptible (DBA/2J) to PR8M [27], [31]. They found that whilst PR8M showed differential pathogenicity in the two strains as previously observed, PR8F and hvPR8 replicated equally well in both strains and caused similar weight loss and mortality, demonstrating that pathogenicity is co-determined by both host and pathogen genetics. Additional studies of the relative sensitivity of the DBA/2J mouse strain compared to C57BL/6 have shown that the DBA/2J strain is susceptible to a wide range of human, avian and swine derived influenza viruses [37], [38].

Boon *et al* explored the genetic determinants of susceptibility to an H5N1 virus containing 7 gene segments of A/Hong Kong/213/2003 H5N1 virus and the PB1 gene segment from A/Chicken/Hong Kong/Y0562/2002 H5N1 (termed HK213) infection using gene mapping of 66 strains of inbred mice (inbred between C57BL/6J and DBA/2J parent strains) and identified five genetic loci (quantitative trait loci 2, 7, 11, 15 and 17) associated with resistance to H5N1 HK213 disease [32]. This suggests that multiple genes determine H5N1 susceptibility in mice. A total of 121 genes located within these five loci were identified as candidates based on RNA expression analysis, which was narrowed to 30 candidates based on differential expression between susceptible and resistant strains. In particular, there were 3, 14, 5, 2 and 6 candidate genes in QTL’s 2, 7, 11, 15 and 17, respectively. The authors compared the outcome of HK213 infection in one mouse strain that expressed hemolytic complement and one that did not, finding that strains expressing hemolytic complement (*Hc*) gene, which is located on QTL 2, experienced increased survival rates at a 10-fold higher initial inoculum. However no association between *Hc* expression and susceptibility to influenza was observed in subsequent work by Trammel or Boon [33], [39].

Boon *et al* further studied the susceptibility of 21 inbred mouse strains to H5N1 HK213 infection, demonstrating that although viral loads were much higher in susceptible strains, the relationship between viral load and cytokine concentrations was the same in resistant and susceptible strains [39]. The authors concluded that this indicates that mouse strain differences in susceptibility to H5N1 lies in a failure to control viral replication rather than the induction of an aberrant inflammatory response. Gene expression and pathway analysis in six strains showed that differential gene expression mostly consisted of up-regulation in susceptible strains of genes in proinflammatory pathways, indicating the immune response is quantitatively but not qualitatively different between strains. Resistant mouse strains (SMR, C57BL/6R, and BALB/cR) did not express a distinctive set of genes controlling replication or disease. 85 individual genes, again mostly associated with proinflammatory pathways, were identified whose expression was associated with susceptibility to severe disease. Three candidate genes identified in the 2009 study were also significant in the 2011 publication and are being further explored (*Grn, Ifi53,* and *Dhx58*). In summary the 2011 work by Boon *et al* suggest that genetic polymorphisms conferring susceptibility to severe H5N1 disease in mice lie in pathways that are involved in the early control of virus replication.

#### Summary of animal models

Mouse models clearly demonstrate a strong genetic effect on susceptibility to a range of influenza viruses. The *Mx* genes are the best studied but their relevance to susceptibility in humans is unknown and although the *MxA* gene should be considered a candidate gene for further studies, there are many other candidates. Crossbred mouse strain studies have identified a large number of potential candidates.

### Familial aggregation or heritability

Independent of genetic effects it is expected that influenza infection will aggregate in families since transmission of influenza is common within households. Family aggregation of severe influenza disease is however more likely to have a direct genetic component but such clustering might also be seen with indirect genetic effects (e.g. genetic predisposition to obesity) or non-genetic shared risk factors (e.g. air pollution).

From the perspective of genetic epidemiology, familial aggregation is said to occur when the frequency of a phenotype is more common amongst close relatives of people with the disease than in the general population [40]. Heritability is the proportion of the variation in the frequency of the phenotype that can be attributed to genetic variation. Familial aggregation can occur without heritability if the increased familial risk is due to shared non-genetic factors. On the other hand, genetics can still be important without any detectable heritability, since if there is no genetic variation in a population then heritability is zero, even though all cases may require a particular genetic background. However, significant heritability does suggest the presence of genetic factors that may be detectable by genotyping studies.

#### Genealogical studies

Two studies utilised large genealogical databases to look for evidence of heritability of susceptibility to death from influenza [41], [42]. The study by Albright *et al* used a Utah database to look at 4855 deaths from influenza between 1904 and 2004 [41]. Gottfredsson *et al* concentrated on the 1918 influenza pandemic in Iceland and looked at 455 deaths over a six-week period [42]. Both studies found evidence of familial aggregation of influenza deaths but differed in their conclusions regarding heritability. Albright *et al* concluded that their results supported heritability since there was an increased relative risk of influenza death amongst relatives of people who died of influenza (relative risk 1.54; 95% CI 1.42-1.67; *P*-value <0.001), and this was greater than observed for relatives of spouses of individuals dying from influenza. Also, influenza deaths in relatives were frequently not associated closely in time (they studied deaths over 100 years) and there was greater than expected relatedness amongst influenza deaths even after close relatives were excluded. Gottfredsson *et al* concluded that their results did not provide evidence of a heritable predisposition to death from 1918 influenza, as they did not identify a statistically significant difference in the relative risk of influenza death in relatives of people who died of influenza (relative risk in 1^st^ degree relatives = 3.75; 95% CI 2.53-5.24) compared to relatives of their spouses (relative risk in 1^st^ degree relatives = 2.95; 95% CI 2.01–4.49. *P*-value for comparison of relative risk in the two groups = 0.198). The apparently conflicting conclusions of these two studies was discussed by Dowell and Bresee, who highlighted the fact that the highest relative risk of influenza death in both studies was in the spouse of cases, so shared social and environmental conditions are important factors and the family aggregation of severe influenza (for whatever reason) offers opportunities to identify and target high risk individuals [43].

The study by Gottfredsson *et al* had ten-fold fewer subjects than the study by Albright *et al* and as such was considerably less well powered to detect differences in the risk of death in relatives of cases compared to relatives of spouses. Also Gottfredsson’s study did not assess the relatedness of cases and was not able to examine deaths outside the six-week period studied, which would be less confounded by common exposures. As such the study by Albright *et al* provides moderate evidence of a heritable component to the risk of influenza death, whereas the Gottfredsson study is inconclusive.

#### Ethnicity

Racial differences in influenza attack rates have been described historically [44], [45]. More recently, an increased risk of hospitalization or death with pandemic influenza H1N1 in indigenous and minority ethnic groups has been reported, particularly in the America’s, Australasia and the Pacific [46]-[51]. Ethnic disparities are observed for many infectious diseases, much of which relates to inequalities in socioeconomic status and related differences in living conditions, access to health care, behaviours, and the prevalence of chronic diseases. No studies have been conducted to determine the genetic component of ethnic differences in rates of influenza hospitalization and death.

#### Familial aggregation of influenza H5N1

Influenza H5N1 is a rare human infection that displays clustering and familial aggregation of cases [3], [5], [52]. Around one third of all H5N1 cases occur in clusters and of the 54 H5N1 clusters summarised in January 2010, 50 were comprised only of blood relatives [5]. Pitzer *et al* have examined the familial aggregation of H5N1 cases andargued that although familial aggregation of H5N1 cases is observed, it is more consistent with non-genetic variation in household risk of exposure to H5N1 than host-genetic factors [53]. Horby *et al* have disputed the inferences drawn by Pitzer *et al* and argued that the totality of the epidemiological data is suggestive of a host genetic effect on susceptibility to H5N1 infection [3]. In addition to the familial aggregation of cases the evidence put forward by Horby *et al* includes: the low number of unrelated clusters, the occurrence of related cases that are separated in time and space (and therefore not compatible with common source exposure), and the poor correlation of exposure with risk [3].

#### Influenza associated encephalopathy (IAE)

Acute encephalitis is a rare but well recognized complication of influenza infection, that occurs mostly in children aged under 5 years and is reported more commonly in East Asia than elsewhere [54]. There is little data to assess if there is genetic susceptibility to IAE other than a report of a mother and daughter with H1N1/09 IAE, two siblings with H5N1 IAE, and an analysis of three IAE cases which reported a missense mutation in the TLR3 gene in one case [55]-[57]. Acute Necrotizing Encephalopathy (ANE) is a distinct clinical syndrome that is characterised by multiple necrotic brain lesions and is associated with influenza infection but also with other viral infections [58]. A subset of patients with recurrent or familial ANE (ANE1) have a missense mutation in the ran-binding protein 2 (RANBP2) gene on chromosome 2 (q12.3) [58]-[60]. The mechanism by which this mutation confers susceptibility to ANE is not yet established. ANE is a very distinct clinical syndrome that, whilst having a genetic basis, is unlikely to have any relation to more general susceptibility to influenza.

#### Summary of familial aggregation or heritability

Although the data are limited and historic, the two genealogy studies clearly demonstrate familial aggregation of the risk of influenza-associated death. The Utah study presents moderate evidence of a heritable component to the risk of influenza-associated death. Whilst familial aggregation of H5N1 cases is generally accepted, there has been no formal estimation of the excess risk in relatives of cases compared to the general population. Such studies (e.g. familial relative risk studies) are theoretically feasible but challenging given the widespread distribution of H5N1 cases in time and space [61]. Estimating heritability of H5N1 is likely to be impossible since it is probably not feasible to disentangle genetic and non-genetic effects with such small numbers of cases.

### Blood group

The 1960’s and 70’s saw a period of interest in the relationship between the ABO blood group and susceptibility to influenza infection. Studies involved observations of natural influenza infection [62]-[64], experimental infection [65], and serological studies [64], [66]-[73]. The data are inconsistent, with authors reporting an increased risk of influenza in subjects with blood group O [62], [65], [66], groups O and B [63], [68], B alone [67], [74], A [73], A and B [64], AB [64], [71], or no difference by blood group [69], [70], [72]. One group examined the ability to excrete soluble ABO blood group antigens in body fluids (secretor) and the risk of respiratory viral infections, and found a positive association between being a ‘secretor’ and influenza A infection [75].

### Human leucocyte antigen (HLA)

Work in the 1970’s by McMichael *et al* and extended by Doherty, Shaw and Biddison demonstrated that cell-mediated lysis of influenza infected cells is dependent on HLA specificities [76]-[81]. It is now well recognised that the HLA molecules plays a central role in antigen presentation to T-cells and indeed *HLA* is the classic example of genetic susceptibility to infectious diseases and of the influence of infectious diseases on human genomes [82]. Subsequent studies in mice and humans demonstrate that the HLA phenotype (H-2 in mice) influences the magnitude and specificity of the cytotoxic T lymphocyte (CTL) response to influenza infection [83]-[85]. Considerable work has also been undertaken to identify particular epitope-HLA molecule combinations that are associated with protective CTL responses in order to inform the design of vaccines targeting cell-mediated immunity [86], [87]. However no genetic studies have been conducted to identify polymorphisms in HLA loci associated with susceptibility to influenza infection. Given the inherent diversity of HLA loci, the complex interaction of *HLA* in determining responses to infection, and the linkage of *HLA* to other genes involved in innate immunity, such studies will be challenging [82], [85].

### Human genetic studies

Only one published human genetic study of susceptibility to influenza was identified. This study was a case control study that included 91 severe H1N1/09 cases and 98 exposed but asymptomatic, unrelated household controls [88]. The authors took a discovery rather than a candidate gene approach, using a commercial chip that incorporates around 50,000 SNPs in regions associated with cardiovascular, metabolic and inflammatory syndromes (HumanCVD Genotyping Beadchip). 28,368 SNPs were analyzed and four SNPs on three different chromosomes had *p*-value *of <0.0001.* These SNPs remained associated after controlling for the potential confounding factors of obesity, diabetes, arterial hypertension, age, gender, and smoking. Three of the SNPs were in genes: an immunoglobulin Fc receptor *(FCGR2A)*; a complement binding protein (*C1QBP);* and a protein that mediates the entry of replication protein A into the nucleus (*RPAIN*). Given the small size of the study, there is a reasonable probability that these are false positive findings, with the false discovery rate (the expected proportion of statistically significant findings that are false positives) for the four SNPs ranging from 22% to 56%.

### Reviews

Five review articles were identified. The review by Trammel and Toth summarized animal and human data on genetic influences on influenza infection, with a particular focus on studies of differential gene expression [89]. This review highlighted the earlier work of Toth *et al* that identified 75 immune related genes (including 13 interferon related genes and 10 chemokine related genes) that were differentially expressed in C57BL/6J compared to BALB/cByJ mice in response to influenza H3N2 HK-X31 infection [35]. The review also identified increased expression of seven common genes in both H1N1/1918 and H3N2 HK-X31 infection of BALB/c mice, and 17 genes that showed increased expression in both human bronchial epithelial cell lines and mice infected with H3N2 (A/Udon/307/72 human bronchial epithelial cell, HK-X31 mice) [35], [90]-[92]. The review by Zhang *et al* proposed a list of around 100 candidate genes that may be related to susceptibility to influenza infection based on existing knowledge of the proteins involved in virus replication and the innate immune response [9]. An Editorial Commentary by Mubareka and Palese on the Utah genealogical study also discussed some potential candidate genes for host susceptibility to influenza, such as mannose-binding lectin, toll-like receptors, retinoic inducible gene I, 2′5′-oligoadenylate synthetase 1, and *MxA* [93]. Horisberger reviewed the data on the relationship between the *Mx1* gene and influenza as it stood in 1995 (see section on *Mx1*) [94]. Horby *et al* reviewed the epidemiological evidence for genetic susceptibility to H5N1 and concluded that the data are suggestive of a host genetic influence on susceptibility to H5N1 disease [3].

## Discussion

In mouse models the severity of influenza infection is clearly associated with both the pathogen and host genome. The observation that similar patterns of susceptibility or resistance of specific mouse strains are observed for a wide range of influenza viruses suggests that some of the host genetic determinants of susceptibility may be common across influenza subtypes. Susceptibility in mice is polygenic, and a number of candidate genes, including *MxA*, have been proposed. To date none of these candidate genes have been tested in studies of humans. Animal experiments will continue to be important for refining understanding of host-pathogen genetic interactions and for testing hypotheses about the pathogenesis of severe influenza.

In humans the best available evidence, relying on a single study of 4855 deaths, suggests a heritable component of susceptibility to death from seasonal and pandemic influenza. Given the numerous confounding factors, replication of this finding will require a similarly large study. Although heritability has not been quantified for H5N1, the marked familial aggregation and other epidemiological features suggest a stronger heritable predisposition. To date only one study of human host genetics and susceptibility to severe influenza has been published and no human genetic polymorphisms associated with susceptibility to seasonal, pandemic or avian influenza have been convincingly demonstrated.

Susceptibility to severe seasonal or pandemic influenza in humans is likely to be polygenic and is also likely to be co-determined by pathogen characteristics, prior infection history, co-morbidities, and environmental factors. In addition, the lack of evidence implicating any specific genes in humans suggests a hypothesis-free genome-wide approach should be taken. As such, very large studies will be required to identify genetic effects on susceptibility to severe influenza.

Pandemic H1N1 offers a rare opportunity to study genetic susceptibility to severe influenza in a context that, compared to seasonal influenza, is less confounded by infection history and pathogen diversity. However, large sample sizes will still be required to detect polygenic traits and case selection will need to consider confounding by cross-protective immunity and co-morbidity. Several groups have compiled series of severe H1N1/09 cases but it seems very unlikely that any single group will have sufficient cases to conduct an adequately powered genome-wide association study [95]. To have a realistic prospect of identifying susceptibility loci for H1N1/09, groups will need to form a consortium, as has been successful for other diseases [96]. The chances of identifying susceptibility loci in H1N1/09 can be enhanced by adopting an ‘extreme-trait’ study design e.g. where cases are previously healthy young adults who develop very severe disease with high viral loads and no evidence of bacterial co-infection. Influenza encephalitis is another ‘extreme-trait’ where case cohorts should be assembled for comparison with other influenza disease cohorts. There may still be possibilities to study susceptibility to 1918 pandemic influenza through linkage studies within large genealogical cohorts, where pedigree and cause of death data stretch back to the early 1900’s [41].

Susceptibility to H5N1 may be less complex than ‘human influenza’, since the phenotype appears to be more dichotomous than continuous, immunity probably plays a lesser role, co-morbidity seems less important, and familial aggregation is more marked. The importance of understanding the pathogenesis of highly pathogenic influenza and the possibility that a rare genetic variant with a moderate to large effect underlies H5N1 susceptibility makes efforts to assemble DNA from H5N1 cases worthwhile. Given the small number of H5N1 cases and the possibility of a rare variant with a moderate to large effect, genome-wide association studies may not be the optimal design and alternative approaches to identifying causal loci may be needed, such as sequencing candidate genes, the whole exome, or the even whole genome [97], [98]. Purely epidemiological studies may contribute to understanding the genetic component of familial aggregation of H5N1 by quantifying heritability.

High viral replication efficiency, or from a host perspective a failure to control virus replication, is emerging as a key factor in severe influenza disease and is determined by both host and virus factors [27], [39]. Thus studies of the determinants of influenza severity may benefit from a combined host-pathogen genetics approach, where the analysis of host genetic associations is conditioned upon the pathogen genotype in order to identify genotype-genotype interactions.

### Conclusion

The fundamental question *‘*Is susceptibility to severe influenza in humans heritable?*’*remains unanswered. It is unanswered not because of a lack of genotyping or analytic tools, nor because of insufficient severe influenza cases, but because of the absence of a coordinated effort to define and assemble cohorts of cases. The recent pandemic and the ongoing epizootic of H5N1 both represent rapidly closing windows of opportunity to increase understanding of the pathogenesis of severe influenza through multi-national host genetic studies.

## Supporting Information

File S1 **Systematic review protocol.**
(PDF)

## References

[1] Sedyaningsih ER, Isfandari S, Setiawaty V, Rifati L, Harun S (2007). Epidemiology of cases of H5N1 virus infection in Indonesia, July 2005-June 2006.. J Infect Dis.

[2] Kandun IN, Wibisono H, Sedyaningsih ER, Yusharmen, Hadisoedarsuno W (2006). Three Indonesian clusters of H5N1 virus infection in 2005.. N Engl J Med.

[3] Horby P, Sudoyo H, Viprakasit V, Fox A, Thai PQ (2010). What is the evidence of a role for host genetics in susceptibility to influenza A/H5N1?. Epidemiol Infect.

[4] Aditama TY, Samaan G, Kusriastuti R, Purba WH, Misriyah (2011). Risk Factors for Cluster Outbreaks of Avian Influenza A H5N1 Infection, Indonesia.. Clin Infect Dis.

[5] World Health Organization (2010). Summary of human infection with highly pathogenic avian influenza A (H5N1) virus reported to WHO, January 2003-March 2009: cluster-associated cases.. Wkly Epidemiol Rec.

[6] Bautista E, Chotpitayasunondh T, Gao Z, Harper SA, Shaw M (2010). Clinical aspects of pandemic 2009 influenza A (H1N1) virus infection.. N Engl J Med.

[7] World Health Organization (2009). WHO Public Health Research Agenda for Influenza..

[8] Konig R, Stertz S, Zhou Y, Inoue A, Hoffmann HH (2010). Human host factors required for influenza virus replication.. Nature.

[9] Zhang L, Katz JM, Gwinn M, Dowling NF, Khoury MJ (2009). Systems-based candidate genes for human response to influenza infection.. Infect Genet Evol.

[10] Lindenmann J (1962). Resistance of mice to mouse-adapted influenza A virus.. Virology.

[11] Staeheli P, Pravtcheva D, Lundin LG, Acklin M, Ruddle F (1986). Interferon-regulated influenza virus resistance gene Mx is localized on mouse chromosome 16.. J Virol.

[12] Arnheiter H, Haller O, Lindenmann J (1976). Pathology of influenza hepatitis in susceptible and genetically resistant mice.. Exp Cell Biol.

[13] Haller O, Arnheiter H, Gresser I, Lindenmann J (1979). Genetically determined, interferon-dependent resistance to influenza virus in mice.. J Exp Med.

[14] Haller O, Arnheiter H, Lindenmann J, Gresser I (1980). Host Gene Influences Sensitivity to Interferon Action Selectivity for Influenza-Virus.. Nature.

[15] Arnheiter H, Haller O, Lindenmann J (1980). Host gene influence on interferon action in adult mouse hepatocytes: specificity for influenza virus.. Virology.

[16] Arnheiter H, Haller O (1983). Mx gene control of interferon action: different kinetics of the antiviral state against influenza virus and vesicular stomatitis virus.. J Virol.

[17] Staeheli P, Danielson P, Haller O, Sutcliffe JG (1986). Transcriptional activation of the mouse Mx gene by type I interferon.. Mol Cell Biol.

[18] Staeheli P, Grob R, Meier E, Sutcliffe JG, Haller O (1988). Influenza Virus-Susceptible Mice Carry Mx-Genes with a Large Deletion or a Nonsense Mutation.. Molecular and Cellular Biology.

[19] Arnheiter H, Skuntz S, Noteborn M, Chang S, Meier E (1990). Transgenic mice with intracellular immunity to influenza virus.. Cell.

[20] Holzinger D, Jorns C, Stertz S, Boisson-Dupuis S, Thimme R (2007). Induction of MxA gene expression by influenza A virus requires type I or type III interferon signaling.. J Virol.

[21] Koerner I, Kochs G, Kalinke U, Weiss S, Staeheli P (2007). Protective role of beta interferon in host defense against influenza A virus.. J Virol.

[22] Haller O, Staeheli P, Kochs G (2009). Protective role of interferon-induced Mx GTPases against influenza viruses.. Rev Sci Tech.

[23] Salomon R, Staeheli P, Kochs G, Yen HL, Franks J (2007). Mx1 gene protects mice against the highly lethal human H5N1 influenza virus.. Cell Cycle.

[24] Tumpey TM, Szretter KJ, Van Hoeven N, Katz JM, Kochs G (2007). The Mx1 gene protects mice against the pandemic 1918 and highly lethal human H5N1 influenza viruses.. J Virol.

[25] Grimm D, Staeheli P, Hufbauer M, Koerner I, Martinez-Sobrido L (2007). Replication fitness determines high virulence of influenza A virus in mice carrying functional Mx1 resistance gene.. Proc Natl Acad Sci U S A.

[26] Dittmann J, Stertz S, Grimm D, Steel J, Garcia-Sastre A (2008). Influenza A virus strains differ in sensitivity to the antiviral action of Mx-GTPase.. J Virol.

[27] Blazejewska P, Koscinski L, Viegas N, Anhlan D, Ludwig S (2011). Pathogenicity of different PR8 influenza A virus variants in mice is determined by both viral and host factors.. Virology.

[28] Zimmermann P, Manz B, Haller O, Schwemmle M, Kochs G (2011). The viral nucleoprotein determines Mx sensitivity of influenza A viruses.. J Virol.

[29] Reeves RH, O'Hara BF, Pavan WJ, Gearhart JD, Haller O (1988). Genetic mapping of the Mx influenza virus resistance gene within the region of mouse chromosome 16 that is homologous to human chromosome 21.. J Virol.

[30] Malo D, Skamene E (1994). Genetic-Control of Host-Resistance to Infection.. Trends in Genetics.

[31] Srivastava B, Blazejewska P, Hessmann M, Bruder D, Geffers R (2009). Host genetic background strongly influences the response to influenza a virus infections.. PLoS One.

[32] Boon AC, deBeauchamp J, Hollmann A, Luke J, Kotb M (2009). Host genetic variation affects resistance to infection with a highly pathogenic H5N1 influenza A virus in mice.. J Virol.

[33] Trammell RA, Liberati TA, Toth LA (2011). Host genetic background and the innate inflammatory response of lung to influenza virus.. Microbes Infect.

[34] Toth LA, Williams RW (1999). A quantitative genetic analysis of slow-wave sleep in influenza-infected CXB recombinant inbred mice.. Behav Genet.

[35] Ding M, Lu L, Toth LA (2008). Gene expression in lung and basal forebrain during influenza infection in mice.. Genes Brain Behav.

[36] Alberts R, Srivastava B, Wu H, Viegas N, Geffers R (2010). Gene expression changes in the host response between resistant and susceptible inbred mouse strains after influenza A infection.. Microbes Infect.

[37] Pica N, Iyer A, Ramos I, Bouvier NM, Fernandez-Sesma A (2011). The DBA.2 mouse is susceptible to disease following infection with a broad, but limited, range of influenza A and B viruses..

[38] Boon AC, deBeauchamp J, Krauss S, Rubrum A, Webb AD (2010). Cross-reactive neutralizing antibodies directed against pandemic H1N1 2009 virus are protective in a highly sensitive DBA/2 mouse influenza model.. J Virol.

[39] Boon AC, Finkelstein D, Zheng M, Liao G, Allard J (2011). H5N1 Influenza Virus Pathogenesis in Genetically Diverse Mice Is Mediated at the Level of Viral Load.. MBio.

[40] Burton PR, Tobin MD, Hopper JL (2005). Key concepts in genetic epidemiology.. Lancet.

[41] Albright FS, Orlando P, Pavia AT, Jackson GG, Cannon Albright LA (2008). Evidence for a heritable predisposition to death due to influenza.. J Infect Dis.

[42] Gottfredsson M, Halldorsson BV, Jonsson S, Kristjansson M, Kristjansson K (2008). Lessons from the past: familial aggregation analysis of fatal pandemic influenza (Spanish flu) in Iceland in 1918.. Proc Natl Acad Sci U S A.

[43] Dowell SF, Bresee JS (2008). Pandemic lessons from Iceland.. Proc Natl Acad Sci U S A.

[44] Armstrong DB (1919). Influenza Observations in Framingham, Massachusetts.. Am J Public Health (N Y).

[45] Isaacs A, Edney M, Donnelley M, Ingram MW (1950). Influenza in an isolated community. An epidemic on Ocean Island.. Lancet.

[46] La Ruche G, Tarantola A, Barboza P, Vaillant L, Gueguen J (2009). The 2009 pandemic H1N1 influenza and indigenous populations of the Americas and the Pacific.. Euro Surveill.

[47] Zarychanski R, Stuart TL, Kumar A, Doucette S, Elliott L (2010). Correlates of severe disease in patients with 2009 pandemic influenza (H1N1) virus infection.. CMAJ.

[48] Thompson DL, Jungk J, Hancock E, Smelser C, Landen M (2011). Risk factors for 2009 pandemic influenza A (H1N1)-related hospitalization and death among racial/ethnic groups in New Mexico.. Am J Public Health.

[49] Centers for Disease Control and Prevention (2009). Deaths related to 2009 pandemic influenza A (H1N1) among American Indian/Alaska Natives–12 states, 2009.. MMWR Morb Mortal Wkly Rep.

[50] Van Kerkhove MD, Vandemaele KA, Shinde V, Jaramillo-Gutierrez G, Koukounari A (2011). Risk factors for severe outcomes following 2009 influenza A (H1N1) infection: a global pooled analysis.. PLoS Med.

[51] Bandaranayake D, Huang QS, Bissielo A, Wood T, Mackereth G (2010). Risk factors and immunity in a nationally representative population following the 2009 influenza A(H1N1) pandemic.. PLoS ONE.

[52] Olsen SJ, Ungchusak K, Sovann L, Uyeki TM, Dowell SF (2005). Family clustering of avian influenza A (H5N1).. Emerg Infect Dis.

[53] Pitzer VE, Olsen SJ, Bergstrom CT, Dowell SF, Lipsitch M (2007). Little evidence for genetic susceptibility to influenza A (H5N1) from family clustering data.. Emerg Infect Dis.

[54] Wang GF, Li W, Li K (2010). Acute encephalopathy and encephalitis caused by influenza virus infection.. Curr Opin Neurol.

[55] Hidaka F, Matsuo S, Muta T, Takeshige K, Mizukami T (2006). A missense mutation of the Toll-like receptor 3 gene in a patient with influenza-associated encephalopathy.. Clin Immunol.

[56] Gonzalez BE, Brust DG (2009). Novel influenza A (H1N1) presenting as an acute febrile encephalopathy in a mother and daughter.. Clin Infect Dis.

[57] de Jong MD, Bach VC, Phan TQ, Vo MH, Tran TT (2005). Fatal avian influenza A (H5N1) in a child presenting with diarrhea followed by coma.. N Engl J Med.

[58] Mizuguchi M, Abe J, Mikkaichi K, Noma S, Yoshida K (1995). Acute necrotising encephalopathy of childhood: a new syndrome presenting with multifocal, symmetric brain lesions.. J Neurol Neurosurg Psychiatry.

[59] Neilson DE, Adams MD, Orr CM, Schelling DK, Eiben RM (2009). Infection-triggered familial or recurrent cases of acute necrotizing encephalopathy caused by mutations in a component of the nuclear pore, RANBP2.. Am J Hum Genet.

[60] Gika AD, Rich P, Gupta S, Neilson DE, Clarke A (2009). Recurrent acute necrotizing encephalopathy following influenza A in a genetically predisposed family.. Dev Med Child Neurol.

[61] Haralambous E, Weiss HA, Radalowicz A, Hibberd ML, Booy R (2003). Sibling familial risk ratio of meningococcal disease in UK Caucasians.. Epidemiol Infect.

[62] McDonald JC, Zuckerman AJ (1962). ABO Blood Groups and Acute Respiratory Virus Disease.. Br Med J.

[63] Frolov VK, Sokhin AA, Sotnik AY, Frolov AK, Lebedinsky AP (1975). Polymorphism of human blood groups and incidence of influenza A/Hong Kong (H3N2).. Acta Virol.

[64] Lebiush M, Rannon L, Kark JD (1981). The relationship between epidemic influenza A(H1N1) and ABO blood group.. J Hyg (Lond).

[65] Tyrrell DA, Sparrow P, Beare AS (1968). Relation between blood groups and resistance to infection with influenza and spome picornaviruses.. Nature.

[66] Potter CW, Schild GC (1967). The incidence of HI antibody to Influenza virus A2/Singapore/1/57 in individuals of blood groups A and O. J Immunol.

[67] Mackenzie JS, Fimmel PJ (1978). The effect of ABO blood groups on the incidence of epidemic influenza and on the response to live attenuated and detergent split influenza virus vaccines.. J Hyg (Lond).

[68] Cuadrado RR, Davenport FM (1970). Antibodies of influenza viruses in military recruits from Argentina, Brazil and Colombia. Their relation to ABO blood group distribution.. Bull World Health Organ.

[69] Evans AS, Shepard DA, Richards VA (1972). ABO blood groups and viral diseases.. Yale J Biol Med.

[70] Potter CW (1969). HI antibody to various influenza viruses and adenoviruses in individuals of blood groups A and O. J Hyg (Lond).

[71] Aho K, Pyhala R, Visakorpi R (1980). ABO associated genetic determinant in H1N1 influenza.. Tissue Antigens.

[72] Watkin IJ, Tills D, Heath RB (1975). Studies of the genetic susceptibility of individuals to infection with influenza viruses.. Humangenetik.

[73] Tyrrell DA, Peto M, King N (1967). Serological studies on infections by respiratory viruses of the inhabitants of Tristan da Cunha.. J Hyg (Lond).

[74] Mackenzie JS, Wetherall JD, Fimmel PJ, Hawkins BR, Dawkins RL (1977). Host factors and susceptibility to influenza A infection: the effect of ABO blood groups and HL-A antigens.. Dev Biol Stand.

[75] Raza MW, Blackwell CC, Molyneaux P, James VS, Ogilvie MM (1991). Association between secretor status and respiratory viral illness.. BMJ.

[76] McMichael AJ, Ting A, Zweerink HJ, Askonas BA (1977). HLA restriction of cell-mediated lysis of influenza virus-infected human cells.. Nature.

[77] McMichael A (1978). HLA restriction of human cytotoxic T lymphocytes specific for influenza virus. Poor recognition of virus associated with HLA A2.. J Exp Med.

[78] Shaw S, Biddison WE (1979). HLA-linked genetic control of the specicity of human cytotoxic T-cell responses to influenza virus.. J Exp Med.

[79] Biddison WE, Shaw S (1979). Differences in HLA antigen recognition by human influenza virus-immune cytotoxic T cells.. J Immunol.

[80] Shaw S, Shearer GM, Biddison WE (1980). Human cytotoxic T-cell responses to type A and type B influenza viruses can be restricted by different HLA antigens. Implications for HLA polymorphism and genetic regulation.. J Exp Med.

[81] Doherty PC, Biddison WE, Bennink JR, Knowles BB (1978). Cytotoxic T-cell responses in mice infected with influenza and vaccinia viruses vary in magnitude with H-2 genotype.. J Exp Med.

[82] Blackwell JM, Jamieson SE, Burgner D (2009). HLA and infectious diseases..

[83] Boon AC, de Mutsert G, Graus YM, Fouchier RA, Sintnicolaas K (2002). The magnitude and specificity of influenza A virus-specific cytotoxic T-lymphocyte responses in humans is related to HLA-A and -B phenotype.. J Virol.

[84] Belz GT, Stevenson PG, Doherty PC (2000). Contemporary analysis of MHC-related immunodominance hierarchies in the CD8+ T cell response to influenza A viruses.. J Immunol.

[85] Day EB, Charlton KL, La Gruta NL, Doherty PC, Turner SJ (2011). Effect of MHC class I diversification on influenza epitope-specific CD8+ T cell precursor frequency and subsequent effector function.. J Immunol.

[86] Wu C, Zanker D, Valkenburg S, Tan B, Kedzierska K (2011). Systematic identification of immunodominant CD8+ T-cell responses to influenza A virus in HLA-A2 individuals.. Proc Natl Acad Sci U S A.

[87] Hertz T, Nolan D, James I, John M, Gaudieri S (2011). Mapping the landscape of host-pathogen coevolution: HLA class I binding and its relationship with evolutionary conservation in human and viral proteins.. J Virol.

[88] Zuniga J, Buendia I, Zhao Y, Jimenez L, Torres D (2011). Genetic variants associated with severe pneumonia in A/H1N1 influenza infection..

[89] Trammell RA, Toth LA (2008). Genetic susceptibility and resistance to influenza infection and disease in humans and mice.. Expert Rev Mol Diagn.

[90] Kash JC, Basler CF, Garcia-Sastre A, Carter V, Billharz R (2004). Global host immune response: pathogenesis and transcriptional profiling of type A influenza viruses expressing the hemagglutinin and neuraminidase genes from the 1918 pandemic virus.. J Virol.

[91] Kash JC, Tumpey TM, Proll SC, Carter V, Perwitasari O (2006). Genomic analysis of increased host immune and cell death responses induced by 1918 influenza virus.. Nature.

[92] Hayashi S, Jibiki I, Asai Y, Gon Y, Kobayashi T (2008). Analysis of gene expression in human bronchial epithelial cells upon influenza virus infection and regulation by p38 mitogen-activated protein kinase and c-Jun-N-terminal kinase.. Respirology.

[93] Mubareka S, Palese P (2008). Human genes and influenza.. J Infect Dis.

[94] Horisberger MA (1995). Interferons, Mx genes, and resistance to influenza virus.. Am J Respir Crit Care Med.

[95] Calafell i Majo F, Gonzalez Candelas F (2011). Genetic factors in severe cases of (H1N1) 2009 influenza.. Rev Esp Salud Publica.

[96] The Wellcome Trust Case Control Consortium (2007). Genome-wide association study of 14,000 cases of seven common diseases and 3,000 shared controls.. Nature.

[97] Cirulli ET, Goldstein DB (2010). Uncovering the roles of rare variants in common disease through whole-genome sequencing.. Nat Rev Genet.

[98] Ng SB, Nickerson DA, Bamshad MJ, Shendure J (2010). Massively parallel sequencing and rare disease.. Hum Mol Genet.

