# Supplementary material for: The Role of Host Genetics in Susceptibility to Influenza: A Systematic Review
Source: PLoS One. 2012 Mar 15;7(3):e33180. doi: 10.1371/journal.pone.0033180 (PMC3305291; doi:10.1371/journal.pone.0033180)
Supplement: File S1 — Systematic review protocol. (PDF) [file pone.0033180.s001.pdf]

Supporting Information file S1: Systematic review protocol

The role of host genetic factors in human susceptibility to influenza infection and disease: a systematic review

Peter Horby, Nhu Y Nguyen, Sarah J. Dunstan, J Kenneth Baillie

Citation

Peter Horby, Nhu Y Nguyen, Sarah J. Dunstan, J Kenneth Baillie. The role of host genetic factors in human susceptibility to influenza infection and disease: a systematic review. CRD Register 2011: N/A

Review question(s)

What is the evidence that host genetic factors play a role in human susceptibility to influenza virus infection and disease?

Searches

Sources

PubMed (includes MEDLINE)
Cochrane Library (CENTRAL)
Web of Science
OpenSIGLE (grey literature bibliographic database)
Reference lists of all studies selected for inclusion will be hand searched to identify further relevant studies
Google Scholar
Consultation with experts in the field

Search limits

English
No limit is placed on date of publication up to the date of execution of the search strategy

Search terms

Pubmed

("Influenza, Human"[Mesh] OR "Influenza A virus"[Mesh]) AND ("Disease Susceptibility"[Mesh] OR (host[All Fields] AND gene*[All Fields]) OR heritab*[ti] OR suscep*[ti] OR famil*[ti] OR polymorphism[ti] OR SNP[ti])

Web of Science

(TS=Influenza OR TS=Influenza A virus) AND (TS=Disease Susceptibility OR (TI=host AND TI=gene*) OR TI=heritab* OR TI=suscep* OR TI=famil* OR TI=polymorphism OR TI=SNP)

The Cochrane Library and OpenGrey

Influenza

Types of study to be included
All study designs are eligible to be included.

Condition or domain being studied
Information on familial clustering of influenza infection and disease in humans.
Information on heritability of susceptibility to influenza infection and disease in humans.
Information on ethnic differences in susceptibility to influenza infection and disease in humans.
Information from in-vitro or in-vivo studies of within species variation in host genetic susceptibility to influenza infection and disease in mammals.

Participants/ population
Studies that contain any data or information related to the domains listed above will be included.
Studies that report only on susceptibility of non-mammalian species (e.g. birds) to influenza infection and disease will be excluded.
Studies that report only on pathogen genetics and susceptibility to influenza infection and disease will be excluded.
Studies that report only on biological mechanisms of susceptibility to influenza infection and disease without reference to host genetics and host genetic variation will be excluded.

Context
Studies in any context will be eligible for inclusion.

Outcome(s)
Primary outcomes
Not applicable
Secondary outcomes
Not applicable

Data extraction (selection and coding)
Two reviewers will run the search strategy independently on the same day. All identified studies, literature or other documents will be screened independently for eligibility by these two reviewers by reviewing the title and abstract. A third reviewer will assess the two independent lists of selected and rejected sources and will make the final selection. The full text of all the sources in the final list will be obtained and reviewed to see if they meet the inclusion/exclusion criteria. The reference list of all selected sources will be reviewed to identify relevant articles that may have been missed by the search strategy. All full text sources will be coded according to the type of information reported in the source. Data and information will be extracted from individual studies using a form that will be piloted prior to its use.

Risk of bias (quality) assessment

It is anticipated that identified studies will be observational epidemiological studies, genetic association studies, narrative review articles, or laboratory based experimental studies.

The quality of observational studies will be assessed by comparison against the checklist produced by the Strengthening the Reporting of Observational Studies in Epidemiology (STROBE) group. The quality of any identified genetic association studies will be assessed against the guidelines produced by the group on Strengthening The Reporting of Genetic Association studies (STREGA). The quality of laboratory based experimental studies will be assessed using a checklist modified from Kilkenny *et al.* [1]. Narrative reviews will not be assessed for quality but will be used as a source for identifying original data for inclusion in the study.

All quality assessments and data extraction will be performed independently by two reviewers, with arbitration of any disagreements by a third reviewer where necessary.

Strategy for data analysis
The characteristics, quality, and any extracted data from included manuscripts will be tabulated. The review will be synthesized in a narrative framework and reported in accordance with the recommendations of the PRISMA working group (Preferred Reporting Items for Systematic reviews and Meta-Analyses).

Dissemination plans
The review will be published in an open-access peer-reviewed journal.

Contact details for further information
Peter Horby
Floor 6, National Hospital of Tropical Diseases
Bach Mai Hospital
78 Giai Phong Street, Dong Da District, Hanoi
Vietnam
peter.horby@gmail.com

Organizational affiliation of the review
Oxford University Clinical Research Unit, Hanoi, Vietnam

Review team
Peter Horby, Oxford University Clinical Research Unit – Wellcome Trust Major Overseas Programme, Hanoi, Vietnam
Nhu Y Nguyen, Oxford University Clinical Research Unit – Wellcome Trust Major Overseas Programme,, Ho Chi Minh City, Vietnam
Sarah J. Dunstan, Oxford University Clinical Research Unit – Wellcome Trust Major Overseas Programme,, Ho Chi Minh City, Vietnam
J Kenneth Baillie, The Roslin Institute, University of Edinburgh, Edinburgh, UK

Anticipated or actual start date
20/06/2011

Anticipated completion date
20/07/2011

Funding sources/sponsors
World Health Organization Global Influenza Programme
Wellcome Trust, UK

Conflicts of interest
None

Language
English

Country
Vietnam

Date of registration in PROSPERO
21st June 2011

1. Kilkenny C, Parsons N, Kadyszewski E, Festing MFW, Cuthill IC, et al. (2009) Survey of the Quality of Experimental Design, Statistical Analysis and Reporting of Research Using Animals. PLoS ONE 4(11): e7824. doi:10.1371/journal.pone.0007824.
